# Essential Elements That Contribute to the Recovery of Persons With Severe Mental Illness: A Systematic Scoping Study

**DOI:** 10.3389/fpsyt.2020.586230

**Published:** 2020-11-19

**Authors:** Atul Jaiswal, Karin Carmichael, Shikha Gupta, Tina Siemens, Pavlina Crowley, Alexandra Carlsson, Gord Unsworth, Terry Landry, Naomi Brown

**Affiliations:** ^1^School of Optometry, Université de Montréal, Montreal, PQ, Canada; ^2^Providence Care, Kingston, ON, Canada; ^3^School of Rehabilitation Therapy, Queen's University, Kingston, ON, Canada

**Keywords:** recovery, rehabilitation, scoping review, elements, mental health, severe mental illness (SMI), outcome, clinical practice

## Abstract

**Introduction:** There is an increasing emphasis on recovery-oriented care in the design and delivery of mental health services. Research has demonstrated that recovery-oriented services are understood differently depending on the stakeholders involved. Variations in interpretations of recovery lead to challenges in creating systematically organized environments that deliver a consistent recovery-oriented approach to care. The existing evidence on recovery-oriented practice is scattered and difficult to apply. Through this systematic scoping study, we aim to identify and map the essential elements that contribute to recovery outcomes for persons living with severe mental illness.

**Methods:** We used the Arksey & O'Malley framework as our guiding approach. Seven key databases (MEDLINE, PubMed, CINAHL/EBSCO, EMBASE, ProQuest, PsycINFO, and Google Scholar) were searched using index terms and keywords relating to recovery and severe mental illness. To be included, studies had to be peer-reviewed, published after 1988, had persons with severe mental illness as the focal population, and have used recovery in the context of mental health. The search was conducted in August 2018 and last updated in February 2020.

**Results:** Out of 4,496 sources identified, sixty (*n* = 60) sources were included that met all of the selection criteria. Three major elements of recovery that emerged from the synthesis (*n* = 60) include relationships, sense of meaning, and participation. Some sources (*n* = 20) highlighted specific elements such as hope, resilience, self-efficacy, spirituality, social support, empowerment, race/ethnicity etc. and their association with the processes underpinning recovery.

**Discussion:** The findings of this study enable mental health professionals to incorporate the identified key elements into strategic interventions to facilitate recovery for clients with severe mental illness, and thereby facilitate recovery-oriented practice. The review also documents important gaps in knowledge related to the elements of recovery and identifies a critical need for future studies to address this issue.

## Introduction

The concept of recovery-oriented care has gained prominence as a philosophical underpinning of the design and delivery of mental health services (1, 2). The recovery approach challenges previously held paternalistic beliefs regarding treatment and prognosis, allowing for a more individualized, holistic approach that respects personal definitions of recovery (3). The literature suggests that recovery is both a process and an outcome, with symptom remission as only one of many possible directions a personal experience with mental illness can take (4). Research has demonstrated that recovery-oriented services are understood differently depending on the stakeholders involved (5, 6). Individuals with mental illness often refer to recovery as a personal transformative journey (7, 8), clinicians discuss recovery in terms of measurable outcomes (9, 10), and decision-makers reference recovery as a vision or guiding philosophy (1). These variations in priorities highlight the lack of common emphasis surrounding recovery as an approach to care, which allows for lack of consistency in the delivery.

The recovery journey is often described as facilitated by a collection of qualities, including holistic, non-linear, and strengths-based, among many others (11, 12). Several theoretical models have been developed that outline characteristics identified in the recovery literature. These frameworks are meant to resolve the lack of clarity that existed previously. The current models contain upwards of five (CHIMES) to 10 (Substance Abuse and Mental Health Services Administration, SAMSHA) components, which are further split into related elements, highlighting aspects such as hope, empowerment, and meaning (10, 13). These models, while helpful as theoretical frameworks, present a challenge in their practical implementation by organizations and clinicians due to a gap between what is often a conceptualization (i.e., hope) and pragmatic capabilities. This gap has allowed providers to advertise recovery-oriented services without necessarily describing what such services entail. The term recovery has the potential to be commandeered by various programs in order to relabel traditional approaches to care (3). Variations in interpretations of recovery lead to challenges in creating systematically organized environments that deliver a consistent recovery-oriented approach to care (2, 6).

In the absence of a pragmatic understanding of recovery, the practical applications may be limited based on the attitude and knowledge of the individual service provider (8, 14, 15). The purpose of this scoping study is to identify and map the essential elements that contribute to recovery for individuals living with severe mental illness. In doing so, we endeavor to create a practical framework that will enable mental health professionals to better understand and incorporate these key elements into their strategic efforts to support clients, in an attempt to narrow the current gap in knowledge translation between knowing and doing.

## Methods

We followed Arksey & O'Malley's scoping review strategy to design and conduct this study (16). This strategy consists of five main steps: (a) identifying the research question, (b) identifying relevant studies, (c) selection of critical articles, (d) reviewing and charting the data, and (e) collating and summarizing the results. This strategy allowed us to identify key concepts, types of evidence, and gaps in the research literature by systematically searching and synthesizing existing knowledge to inform mental health care practice. We also incorporated recommendations of Peters and colleagues for systematic scoping review by reporting the operational definition of “population,” “concept,” and “context” of the review, and providing information on search strategy, inclusion criteria, and data synthesis (17).

### Identifying the Research Question

The following research question guided this systematic scoping study: What are the essential elements that contribute to recovery outcomes for individuals living with severe mental illness? For this study, the population was individuals with severe mental illness. Severe mental illness was defined as “a mental, behavioral, or emotional disorder resulting in serious functional impairment, which substantially interferes with or limits one or more major life activities” (18). We defined the concept of recovery as “… *a deeply personal, unique process of changing one's attitudes, values, feelings, goals, skills, and roles. It is a way of living a satisfying, hopeful, and contributing life even with limitations caused by illness*” [11, p. 15]. The spatial and temporal context for this review is studies from across the globe that are related to the recovery of individuals with severe mental illness and were produced from 1988 (one of the first published articles to reference “Recovery” as a concept) (19).

### Identifying Relevant Studies

To identify pertinent journal articles, we developed our search strategy in consultation with a health sciences research librarian. We used seven key databases, including MEDLINE, PubMed, CINAHL/EBSCO, EMBASE, ProQuest, PsycINFO, and Google Scholar, to locate the relevant literature. The keywords used to identify relevant studies are presented in Box 1. Please note that these keywords varied to some extent depending on the different indexing schemes of respective databases. The search was conducted in August 2018 and last updated in February 2020.

Box 1 **Search terms.**

(severe mental illness OR chronic mental illness OR serious mental illness OR persistent mental illness OR psychosis OR schizophrenia OR bipolar disorder OR depression OR personality disorder OR trauma disorders OR anxiety) AND (recovery OR psychosocial rehabilitation OR psychiatric rehabilitation) AND (theor$ OR framework OR model OR dimension OR paradigm OR concept$ OR frame of reference OR approaches OR oriented services OR oriented interventions OR themes OR processes OR outcomes)

### Study Selection

**Figure 1:**
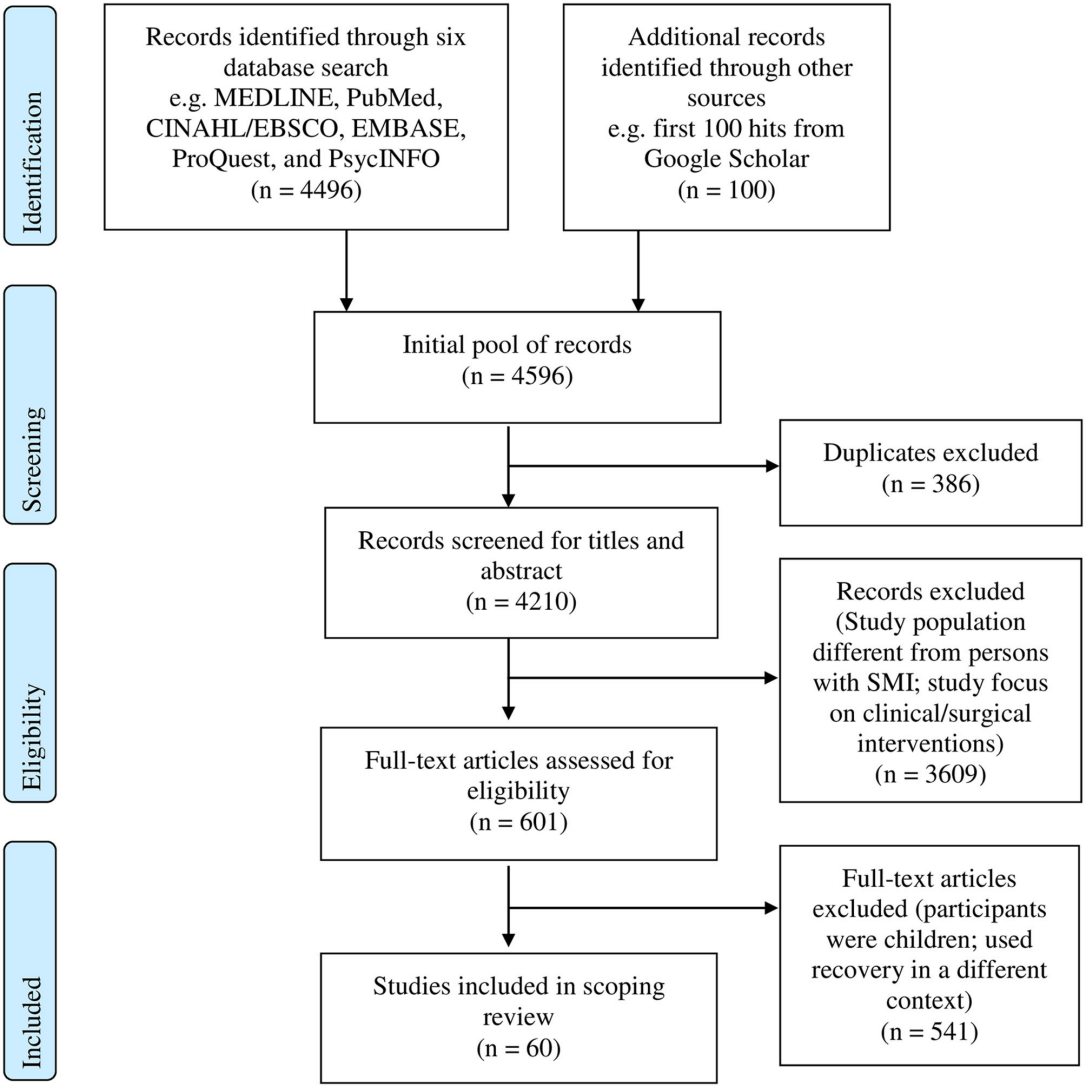
PRISMA flow chart. SMI, Severe mental illness. Source: (20).

We applied inclusion and exclusion criteria to the studies that emerged in the initial search, as documented in Table 1. We followed a two-stage screening process to select studies that matched our objective. The first stage involved reading the titles and abstracts, and the second stage included reading the full-text articles. Two independent reviewers screened the titles and abstracts, and the selected articles were divided within the research team for full-text review. Any discrepancies were resolved during the monthly consultation meetings of the research team. The final list of articles was compiled into an MS–Excel file/spreadsheet for data charting. The information on a number of sources identified, screened, found eligible, and finally included in the study is presented in the PRISMA flowchart (Figure 1). The PRISMA 2009 checklist can be found in the Supplementary Material.

**Table 1:** Inclusion and exclusion criteria.

**Included when:**	**Excluded when:**
• Specified study population: adults with severe mental illness or chronic mental illness or serious mental illness or persistent mental illness or psychosis or schizophrenia or bipolar disorder or depression or personality disorder or trauma disorders or anxiety. Comorbid conditions, in addition to the conditions listed, were acceptable. • Were produced from 1988 (Article by Pat Deegan- The Lived Experience of Rehabilitation was one of the first published articles to reference “Recovery”)	• Related to children with severe mental illness. • The article focused solely on clinical (medicine-related) or surgical interventions, dementia or intellectual impairment or developmental disability or learning disability or substance use or substance abuse or addictions or substance-induced psychosis or clinical condition induced delirium
• Were peer-reviewed articles.	• Full text not available in the English language
• Were focussed on aspects of recovery, psychosocial rehabilitation, or psychiatric rehabilitation in the mental health field	• Used recovery in a context other than mental health

### Data Charting

The descriptors used for data charting included authors, journal title, time and location of the study, study design, study population, sample size, the purpose of the study, key outcomes or results, and any other data relevant to our study objectives. The descriptor “not available” was used if any of the required information was missing from the source. All the authors completed data charting in the spreadsheet.

### Data Synthesis and Reporting

After data charting was completed, the research team prepared a descriptive numerical summary and conducted a qualitative thematic analysis to present the key findings of the study. A summary of descriptive findings was collated from the spreadsheet, and each team member coded them independently using Braun and Clarke principles of thematic analysis (21). Later, all team members listed their codes and similar codes were clustered to key themes inductively in two consecutive team meetings. Details on study design, study population, sample size, time and location, and purpose of the study are given in the form of numerical summary, while critical results and outcomes are reported in the way of thematic synthesis. We are reporting this study using the Preferred Reporting Items for Systematic reviews and Meta-Analyses extension for Scoping Reviews (PRISMA-ScR) Checklist (22).

## Results

Out of 4,496 sources identified, 601 references were extracted from seven bibliographic databases. Sixty (*n* = 60) sources were finally accepted that met all the selection criteria.

### Characteristics of the Records Included in the Study

Of the 60 sources that were included in the final review, the majority were empirical, comprising qualitative studies (*n* = 32), followed by quantitative (*n* = 21), and mixed methods (*n* = 3). The non-empirical records were primarily literature reviews (*n* = 4). The records presenting empirical research covered a broad spectrum of methodologies (e.g., quantitative, qualitative, and mixed), designs (e.g., longitudinal, cross-sectional, randomized controlled trial, systematic reviews, and case study) and data collection strategies (e.g., interviews, focus groups, ethnographic observations, and surveys). Table 2 provides details on study location, study type, participant population, sample size, and study focus.

**Table 2:** Characteristics of records included in the study (*n* = 60).

**References**	**Location of study**	**Study type**	**Study population**	**Sample size**	**Focus of article**
Aldersey and Whitley (23)	Canada	Qualitative Study	Adults with a diagnosis of severe mental illness	54	Perceived barriers and facilitators to recovery related to family
Andresen et al. (24)	Australia	Literature review	Mental health consumers	36 articles	Meaning of recovery through client experience
Anthony (25)	USA	Qualitative study	Individuals who had a self-reported diagnosis of severe mental illness	10	Experiences of helping partnerships that facilitate recovery
Bonfils et al. (26)	USA	Randomized controlled trial	Individuals with Schizophrenia	45	Recovery and how clients' words reflect hope
Bonfils et al. (27)	USA	Quantitative study	People with schizophrenia spectrum disorder, bipolar disorder and major depressive disorder	167	Association between parenthood and recovery
Borg and Kristiansen (28)	Norway	Qualitative study	Persons with severe mental illness	15	Recovery-oriented professionals
Chinman et al. (29)	USA	Qualitative study	Three clients served at the Connecticut Mental Health Center	3	Understanding the most useful aspects of ACT teams for recovery
Connell et al. (30)	Australia	Qualitative study	Young adults (ages 19–24) following the first episode of psychosis	12	The extent to which a single psychotic episode diminishes self
Davidson et al. (31)	USA, Italy, Norway, Sweden	Qualitative study	Individuals who have experienced recovery from psychosis	12	Role of various factors in processes of recovery
Firmin et al. (32)	USA	Mixed method study	Adults diagnosed with schizophrenia-spectrum disorders	46	Helping the behaviors of those diagnosed with SMI
Forchuk et al. (33)	Canada	Qualitative study	Individuals with symptoms of psychosis	10	Changes in perceptions of recovery with time
Giusti et al. (34)	Italy	Quantitative study	Inpatient adults diagnosed with schizophrenia	76	Predictors of recovery
Griffiths et al. (35)	UK	Quantitative study	Adults with diagnoses including depression, schizophrenia, bipolar disorder, personality disorder, and anxiety disorder	181	Examining recovery after a person moves from an inpatient psychiatric setting into a residential program
Gumley and Macbeth (36)	UK	Quantitative study	Individuals with psychosis	29	Development of a narrative-based measure of compassion concerning recovery
Hamm et al. (37)	Australia	Mixed method study	Patients in primary care experiencing depressive symptoms	564	Role of inner resources (primarily resilience) in the recovery of depressive symptoms
Hancock et al. (38)	Australia	Qualitative study	Adults with severe mental illness enrolled in a recovery program	13	Understand early-stage mental health recovery experiences
Hasson-Ohayon et al. (39)	Israel	Quantitative study	Persons with a diagnosis of serious mental illness	107	Association between insights and recovery
Hasson-Ohayon et al. (40)	Israel	Quantitative study	Adults diagnosed with schizophrenia or schizoaffective disorder	80	The connection between having a sense of meaning and recovery
Hoffmann and Kupper (41)	Switzerland	Quantitative study	Individuals with schizophrenia in the vocational rehab program	75	Psychosocial recovery for schizophrenia
Hungerford and Richardson (42)	Australia	Qualitative study	Caregivers	10	Family engagement and recovery
Hyde et al. (43)	Australia	Qualitative study	Patient with mental illness	8	Consumers' lived experience of inpatient care
Jerrell et al. (44)	USA	Quantitative study	Individuals with schizophrenia, depression, bipolar disorder, schizoaffective disorder etc.	459	Meaning and elements of recovery; and psychometric elements to measure recovery
Jorgensen et al. (45)	Denmark	Quantitative study	Individuals with schizophrenia	101	Relationship of subjective elements and objective elements of recovery.
Jose et al. (46)	India	Systematic review	Schizophrenia	25 studies	Consumer perspectives on recovery from Schizophrenia
Kidd et al. (47)	Canada	Qualitative study	Racialized women with severe mental illness	6	The intersection of gender and ethnicity with the recovery from mental illness
Kilbride and Pitt (48)	UK	Qualitative study	Persons with psychosis	7	Process of recovery
Kwok (49)	Canada	Qualitative study	Bipolar disorder	1	Limitations of the clinical model of recovery
Lakeman (50)	Ireland	Quantitative study	Experts by experience	31	Recovery focussed competencies
Liberman et al. (51)	USA	Qualitative study	People with schizophrenia	55	Operational definitions of recovery
Liberman and Kopelowicz (52)	USA	Narrative study	People with schizophrenia	Not applicable	Elements of recovery
Markowitz (53)	USA	Quantitative study	Persons with mental illness in consumer-run self-help groups and outpatient settings	610	Examine social-psychological components in the recovery process
Mezzina et al. (54)	Italy	Qualitative study	Persons with psychosis	Not reported	Role of social factors in recovery from psychosis
Mihaljevic et al. (55)	Croatia	Quantitative study	Adults in inpatient or outpatient treatment for a depressive episode	99	Association between depression and spirituality
Murphy (56)	Not reported	Qualitative study	Individuals with serious mental illnesses	8	Meaning of recovery from psychosis
Myers (57)	USA	Qualitative Case study	Persons with schizophrenia	1 organization	Recovery-based mental health care
Nasser and Overholser (58)	USA	Quantitative study	Psychiatric in-patients with major depression	62	Potential benefits of support from family, friends, and spiritual beliefs
O'Keeffe et al. (59)	Ireland	Qualitative study	Individuals with first episode psychotic disorders	20	Experiences of service utilization and suggestions for change to improve recovery
Ouwehand et al. (60)	Netherlands	Qualitative study	Patients with bipolar disorders	10	Interpretation of religious and spiritual experiences during mania, depression and recovery
Park and Sung (61)	South Korea	Quantitative study	Individuals with Schizophrenia	60	Effects on helplessness and recovery of an empowering program for patients with schizophrenia
Ringer et al. (62)	USA	Quantitative study	Patients with schizophrenia	78	Subjective indicators of recovery
Rosa et al. (63)	Spain	Quantitative study	Individuals diagnosed with bipolar disorder	119	Functional recovery in two samples of people with bipolar disorder
Rouse et al. (64)	Canada	Qualitative study	Individuals with severe mental illness and organizational staff	78	Elements of recovery : Mechanisms and outcomes
Rudnick (65)	Canada	Literature review	Individual diagnosed with schizophrenia	Not reported	Philosophical framework on Essentials to recovery
Sapani (66)	UK	Literature review	Mental health staff and consumers	Not reported	Examine recovery and what principles are utilized in practice
Schön (67)	Sweden	Qualitative study	Adults diagnosed with psychosis, bipolar disorder and personality disorder	30	Understanding recovery from gender perspective
Schreiber (68)	Canada	Qualitative study	Women with Depression	70	Impact of depression for women
Sells et al. (69)	USA	Qualitative study	Individuals with severe mental illness	Not reported	Arenas of recovery
Shahar et al. (70)	UK	Quantitative study	People diagnosed with schizophrenia spectrum disorders	105	Role of dependency, self-criticism and efficacy in recovery
Thomas and Salzer (71)	USA	Quantitative study	Adults with serious mental illnesses	46	Correlation of peer-to-peer relationship with recovery-oriented outcomes
Tooth et al. (72)	Australia	Qualitative study	Individuals with schizophrenia	57	A consumer perspective on recovery from schizophrenia
Topor and Denhov (73)	Sweden	Qualitative study	Individuals with severe mental illness	58	Role of others in recovery
Torgalsbøen (74)	Norway	Quantitative study	Individuals with schizophrenia	50	Elements contributing to the recovery
Torgalsbøen and Rund (75)	Norway	Mixed method study	Individuals fully recovered from schizophrenia	6	Course and outcome of schizophrenia.
Tsai (76)	USA	Qualitative study	Individual with serious mental illness	1	First-hand experience of recovery
Tse et al. (77)	Hong-Kong	Quantitative study	Adults with bipolar disorder in remission	75	Psychosocial correlates of recovery stag
van Grieken et al. (78)	Netherlands	Qualitative study	Adults who recently recovered from depression	20	People's effort to recovery from depression
Warwick et al. (79)	UK	Qualitative Study	Adults previously diagnosed with bipolar disorder	12	Processes underlying recovery
Whitley (80)	Canada	Qualitative study	Adults living with severe mental illness	47	Relationship between ethnicity, culture, and recovery.
Williams and Collins (81)	Canada	Qualitative Study	Individuals with schizophrenia	15	Subjective experience of schizophrenia and recovery
Wood et al. (82)	UK	Q-methodology (literature review followed by qualitative interviews)	Individuals with psychosis	40	Recovery from psychosis

Almost all of the sources stemmed from high-income countries. Many studies were conducted in the continent of North America (*n* = 25) [United States of America (*n* = 16) and Canada (*n* = 9)], Europe (*n* = 16), Australia (*n* = 7), and the United Kingdom (*n* = 7) followed by two studies from Israel, one from India, one from South Korea, and one from China. Just under half of the included articles (*n* = 27, 45%) were published before 2010 (1999–2010), while 55% (*n* = 33) were published after 2010 (2011–2019). Forty-seven percent of included studies (*n* = 28) were published within the last 5 years.

Of the total studies, 42% focused broadly on severe mental disorders (*n* = 25). Most did not specifically mention individual diagnoses to protect the privacy of their participants. A number of sources (32%) focused on or had participants diagnosed with schizophrenia or schizophrenia spectrum disorders (*n* = 18), followed by depression (*n* = 5), psychosis (*n* = 9), and bipolar disorders (*n* = 5). Of the 60 studies included, 95% of studies (*n* = 57) included direct perspectives of individuals with severe mental illness, while three studies focused on the caregiver, expert, and staff experiences, respectively.

The majority of studies (*n* = 33, 55%) focused broadly on meaning, elements, or aspects of recovery for individuals with severe mental disorders. Some studies (*n* = 20, 33%) focused on the relevance of specific elements such as hope, resilience, self-efficacy, spirituality, social support, race/ethnicity etc. and their association with the processes underpinning recovery. A few studies (*n* = 8, 13%) examined various aspects of treatment approaches directed toward recovery.

### Thematic Analysis/Qualitative Synthesis

**Figure 2:**
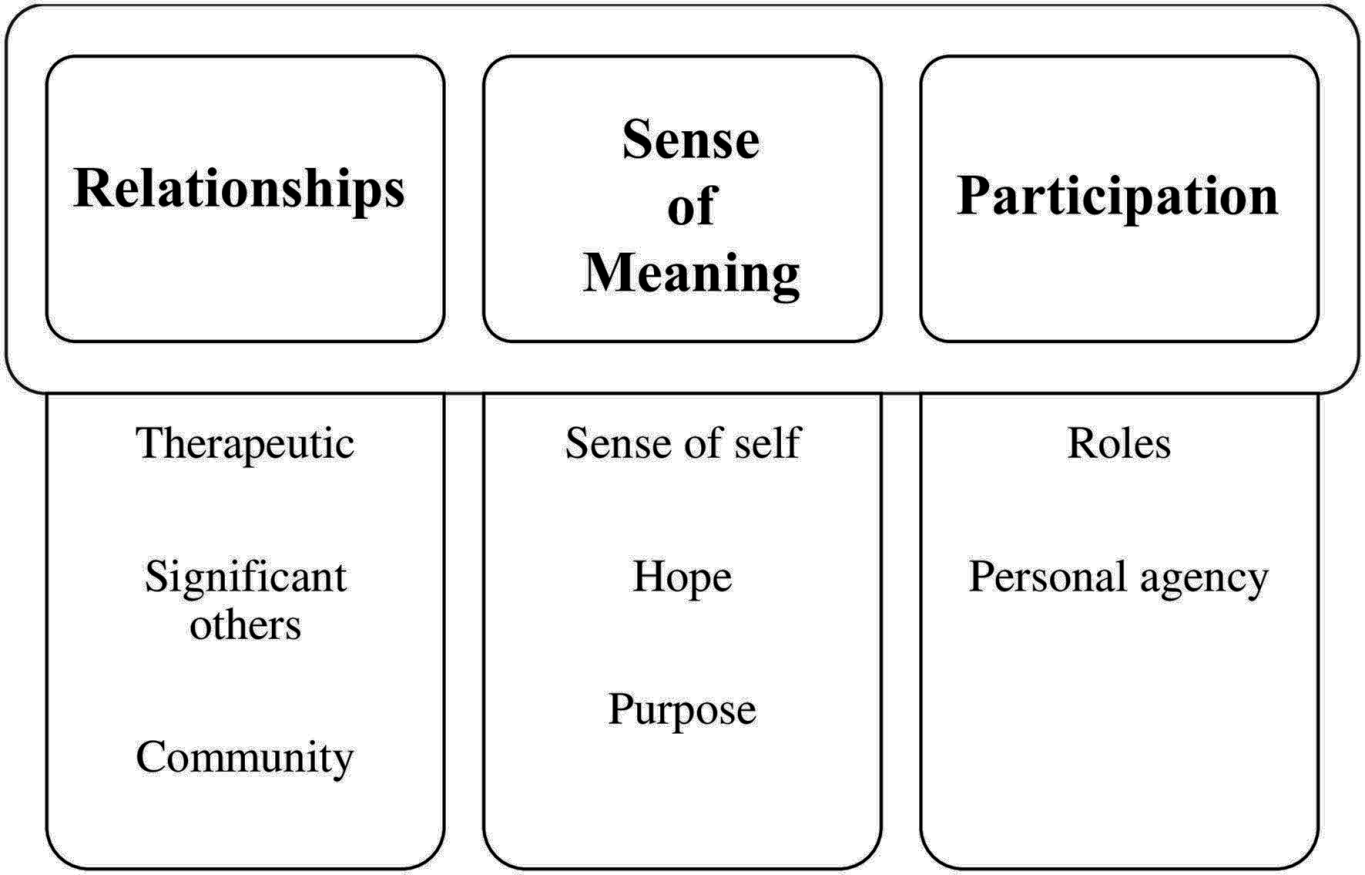
Key themes—elements of recovery.

Through qualitative analysis of the data (see Methods section), the research team developed consensus on the three main elements contributing to recovery from severe mental illness: relationships, sense of meaning, and participation (Figure 2). The research team also reached a consensus on eight sub-elements within these three core elements. Each of the elements and sub-elements is discussed here:

#### Relationships

A number of studies (*n* = 41, 68%) highlighted the importance of supportive relationships in facilitating recovery from severe mental illness. Our analysis generated three relationship subthemes: therapeutic relationships, relationships with significant others, and relationships with the broader community. Please note that these three categories were not mutually exclusive, and each had substantial overlaps.

##### Therapeutic Relationships

Several studies included in our review (*n* = 17, 27%) reported that relationships with service providers impacted the experience and extent of recovery in individuals with severe mental illness (25, 28, 35, 37, 54, 69, 73, 76, 82). Individuals perceived therapeutic relationships, characterized by human qualities such as an attitude of equality, acceptance, empathy, respect, compassion, connection, collaboration, safety and confidence, as helpful in their recovery from schizophrenia (28, 59, 64, 75, 83). Studies also emphasized the role of the therapeutic relationship in kindling and sustaining hope as one of the major factors contributing to full recovery for persons diagnosed with severe mental illness (32, 75). In one model of recovery, clients considered strong and trusting relationships (between service providers, themselves, and their families) that supported their navigation of the mental health system, as essential to their improved mental health (29). Similarly, participants from another study described relationships with clinicians as more facilitative of recovery than the treatment being offered (67). For men, the perceived expertise of the professional and a sense of reciprocity were most important, while for women, trust, listening, and emotional support were more facilitative of recovery (67).

##### Relationship With Significant Others (*Friends/Family*)

Several studies (*n* = 10, 16%) found that reconnecting with family and friends was integral to the process of recovery for individuals with severe mental illness (33, 34, 43, 74). One study described the fostering of relationships, facilitated by opportunities to interact with others, develop social skills, and reduce isolation through building social networks, as a core mechanism of recovery (64). In a study involving individuals with depression, participants identified that remaining socially engaged with friends, family, and colleagues, who were informed of the impact of their experience of depression, was key to obtaining the support needed for recovery (78). Similarly, a few studies identified supportive family members or caregivers, who encouraged and positively reinforced the incremental progress of the individual, and were involved as per the choice of the individual, as a critical factor in long-term recovery outcomes for people with schizophrenia (23, 52). A study comparing support from friends and family found that support from friends or others outside of the family network may facilitate recovery from depression more than support from family, as participants' perceived family members as obligated to provide support (58). Thus, perceptions of support from friends or family may influence the recovery process differently (58). Another study found an association between interpersonal relationships, characterized by secure attachment, and participants' levels of hope and self-esteem, suggesting that secure attachments are related to recovery (62).

##### Relationship With the Broader Community

The research team identified one's relationship with the broader community as the third type of relationship critical to recovery from mental illness, as reported in 25% of the included studies (*n* = 15) (35, 40, 43, 54, 78, 81). Two studies described recovery as an interactive social journey, involving meaningful, inclusive social relationships, within which individuals exercise rights, encounter opportunities, and receive responses that either support or fail to support their social needs (54, 81). This study described peer support as a bridge toward social opportunities within the wider community and identified the sense of fellowship peer support provides as supportive of recovery (64). Other studies similarly identified peer relationships as key to supporting recovery (61, 72). Many studies described connecting to others, social functioning, and social relationships as important for recovering “coherence,” reducing isolation, making meaning of experiences, and instilling hope (35, 58, 62, 82). One study, involving a group rehabilitation program, described a sense of belonging, or security, acceptance, and connection to others that fosters a feeling that one is a part of a stigma-free community as a mechanism of recovery (64). Another study similarly identified stigma as a factor that impeded recovery through interfering with social inclusion in the community (72).

#### Sense of Meaning

Majority (*n* = 49, 82%) of the included studies described a sense of meaning as a facilitator of recovery from severe mental illness. During the qualitative synthesis stage, the research team divided a sense of meaning into three key elements: sense of self, hope, and purpose.

##### Sense of Self

Just under one-third (*n* = 18, 30%) of the included studies identified a “sense of self” as making an important contribution to recovery. However, each of these studies defined and examined “sense of self” differently. For example, one study involving individuals with severe mental illness (*n* = 107), suggested that enhancing a positive, clear, non-stigmatizing sense of self may lead to a positive recovery process (39). In a study that examined the experience of recovery from psychosis, recovery was seen as non-linear, occurring in stages, and encompassing physical, emotional, mental, and spiritual aspects of the person (33). Other studies found that building self-efficacy, self-sufficiency, self-acceptance, and reducing self-stigma was a critical recovery mechanism that involved helping individuals gain skills and feel more capable of, and confident in, acting independently and participating in society (31, 32, 40, 46, 64, 70, 79, 81, 84).

Studies seeking individuals' perspectives on recovery from severe mental illness identified personal agency as a key to recovery (41, 72, 78, 79). Factors described as contributing to personal agency included perceived determination to get better, optimism, taking responsibility to help themselves, and understanding, managing, and accepting their illness (41, 44, 72, 79). Another study described self-organization, an ongoing process of “self-creation and self-repair,” as central to recovery in schizophrenia [(56), p. 273]. Participants from another study identified rebuilding the self through self-awareness and reconciling with the past as one of the important components of recovery (77). Other studies identified the acquisition of skills for daily living and self-management as contributing to recovery outcomes (55, 64).

##### Hope

A number of studies (*n* = 21, 35%) identified hope as a strong determinant of recovery (24, 26, 34, 40, 41, 43, 48, 77). Included studies conceptualized hope in different ways. Many studies described hope in terms of spirituality. Studies described faith as helping to generate and maintain hope in recovery and as providing comfort throughout the process (56, 75). A quantitative study, involving 99 patients with depression, identified higher spirituality as a stronger predictor of recovery (55). This study defined spirituality as a personal quest for a sense of purpose and meaning of life, rather than as religious affiliation. It identified domains of “wholeness and integration,” “inner peace,” and “hope and optimism” as the strongest contributors to the negative association between spirituality and depression (55). Other authors reported similar findings involving different populations (38, 46, 52, 67, 72, 74). For instance, studies involving a group of individuals diagnosed with psychosis or schizophrenia-spectrum disorders identified hope, along with a sense of self-agency, wellbeing resilience, and strength, as integral components of recovery (26, 30, 32, 38, 46).

##### Purpose

Several studies (*n* = 10, 17%) identified a “sense of purpose” as an element that contributes to recovery (24, 40, 43). Individuals with a severe mental illness described the “sense of purpose,” generated from participating in the running of a clubhouse recovery program and contributing to shared goals, as a mechanism of recovery (64). Another study identified creating a sense of purpose as the most important aspect of recovery (48). This study, along with other studies, suggested that the development of self-esteem, agency, and active participation in life is an empowering process that both creates and is created by a sense of purpose (32, 46, 48, 53, 61). One study described empowerment as a gendered recovery process (67). It found that women described recovery as a process of regaining their “whole” identity, moving from a sense of oneself as the object of treatment to the perception that one is a subject, engaged in, and accepting of, the recovery process [(38), p. 563].

#### Participation

The research team divided participation into two sub-themes: roles and personal agency. Articles grouped within this theme described participation in meaningful roles within one's family and community (roles) and participation in one's life choices (agency) as critical elements contributing to recovery. Just under half (*n* = 25, 42%) of the included sources identified participation as an important recovery theme, of which 17 studies highlighted roles and eight studies highlighted personal agency.

##### Roles

A number of studies (*n* = 17, 28%) found certain roles to be associated with recovery from severe mental illness. Several studies described meaningful, helping, or productive roles as positively impacting the recovery journey of patients with severe mental illness (27, 32, 46, 76, 77, 82). Some of these studies explored the different life roles of their participants related to productive work such as employment, parenthood, volunteering, religious practice, or self-care (35, 46, 57, 60, 64, 66, 72, 78, 80). For example, one study found that gaining and maintaining employment was associated with financial stability, increased self-esteem, and empowerment (66). Decreased boredom, associated with employment, was also associated with an increase in meaningful activities, which was, in turn, associated with increased social interaction and feelings of inclusion (66).

A few studies also looked at the interplay of gendered roles, culture, and ethnicity and their influence on recovery (47, 49). A study examining the importance of enabling women to challenge assumptions related to roles, limitations, and rules considered this process as empowering women to make sense of their depression and to construct new lives (68). However, in another study, the authors argued that traditional gender roles advantaged women (67). The authors of this study found that greater acceptance of women as dependent on social supports, such as family, and reduced pressures for women to work and study, actually lessened the burden of role expectations and contributed toward recovery (67). In a qualitative study exploring the relationship between ethnicity, culture, and recovery (*n* = 47), all the ethnic groups identified progress in employment, social engagement, and community participation as facilitators to recovery while identifying stigma, financial constraints, and psychiatric hospitalization as barriers to recovery (80).

##### Personal Agency

Several studies (*n* = 8, 13%) identified active agency in one's recovery path, cultivated through opportunities to take an active role in treatment decisions and to choose to use services according to one's wants and needs, as a critical element of recovery from severe mental illness (31, 34, 35, 64, 76). One study referred to the personal agency as “self-directed empowerment” in discussions regarding the recovery of study participants with bipolar disorder (77). In a study on self-management in the recovery from depression, participants found that assuming an active and critical attitude toward the illness and service providers and using self-management strategies in their daily life such as goal setting, activity schedules, to-do lists, and distractions contributed to their recovery (78). In another study, participants shared that autonomous action helped them to become independent citizens rather than subjects of a paternalistic mental health care system (57).

## Discussion

This systematic scoping review aimed to identify and explore the essential elements of recovery to better guide practical clinical interventions. The authors approached this research through a functional lens with a focus on the practical application of theoretical knowledge to better support evidence-informed delivery of care. Previous research has found the boundary between the questions, “what constitutes recovery and what are the factors that enhance it” are blurry [(85), p. 177]. The themes generated in this scoping review represent an ongoing fusion of recovery as a means and an end, suggesting that recovery can be promoted through enhancing relationships, sense of meaning, and participation, and also be measured through the presence of each of these elements in our lives.

It is noteworthy that most of the literature included qualitative studies conducted with individuals with severe mental illness belonging to North American countries. Within the studies included, schizophrenic disorders were dominantly represented, with only a few studies focusing on depression, which is among the largest single cause of disability worldwide (83). Similarly, only a few studies included the perspectives of informal caregivers/support persons or professionals working with clients with severe mental illness (24, 42). These findings point to the fact that while there has been an increase in the effort to understand recovery from client perspectives, there remains a need to incorporate the diversity in perspectives and socio-demographic needs of those living with a broader spectrum of severe mental illness and those offering support.

The research team acknowledges that recovery is a deeply personal and unique journey for each individual, which implies that each person may have their own definition of what recovery entails for them [(12), p. 1250]. Despite the personal nature of recovery, previous research has identified several elements commonly cited in the literature as influential to recovery. Our findings on recovery compliment and simplify recovery components discussed in this research, including those described in a recent systematic review specific to recovery and mental illness (2). Our research team grouped common elements found in the literature into three pillars: relationships, sense of self, and participation. The significance of each pillar and its respective components allow for infinite variation in the recovery journey while providing clinicians with a practical approach to supporting those individuals. These pillars and elements do not represent an exhaustive list but are consistent themes throughout the literature and can offer clinicians guidance in translating recovery theory into practice.

Keeping the three pillars in the forefront of the clinical practice, while allowing the client to define their specific recovery journey, may provide clinicians with a pragmatic approach to better facilitate client recovery. This research team also feels that the simplistic approach of the three pillars (relationships, participation, and sense of self) to this complex topic will enable dynamic discussions on this issue with clients, support members and also with policymakers. Demonstrating a clear need to create an environmental shift through policy to better support personal recovery through the utilization of pragmatic relatable terms may help to move the recovery model forward regardless of objective outcome measures.

The nature of recovery is unique and often ambiguous, which presents an ongoing challenge to clinicians on how to best facilitate/support the recovery process. System-level policies and funding models emphasize measurable outcomes, but the personal recovery journey does not always lend itself to measurable change. This sanctions the traditional paternalistic approach in which clinicians and family members are seeking symptom remission despite what the recovery literature suggests that the client is not necessarily focused on remission. The traditional approach is symptom remission through medication in order to permit participation, engage in relationships and deepen one's sense of self. Perhaps if the focus is weighted more heavily on the pillars of recovery, symptom reduction would be the outcome. To challenge this traditional approach would also require environmental/system-level changes to allow for appropriate supports to be available without the necessity of traditional outcome measures.

Underemphasized, in the included articles, was the role that environmental interventions could play in recovery. This predominant focus on person-level elements has likewise been noted in a recent systematic review and could be explained, in part, by accepted definitions of recovery that do not explicitly reference the environment as a site of change (2, 7, 11, 19). Despite the tendency in clinical practice to direct service toward the individual, a growing body of research shows that the environment is often more immediately amenable to change than the person (86–90). The WHO's Commission on the Social Determinants of Mental Health supports the role of the environment in promoting recovery, arguing that mental health is shaped “to a great extent by the social, economic, and physical environments in which people live” [(91), p. 8]. Many models, such as the WHO International Classification of Functioning, Disability and Health (ICF), the Person-Environment-Occupation (PEO), the Canadian Model of Occupational Performance and Engagement (CMOP-E), and Ecology of Human Performance, also recognize the environment as a valid and consequential site for recovery-oriented intervention (92). Though treating the environment as an agent of change is becoming more common in policy and public health initiatives seeking to create recovery opportunities for clients, such as Housing First and Individual Placement and Support (93, 94), access to these services is often limited (3). This scoping review reinforces that essential elements of recovery must be identified through a broader lens that considers the role of micro, meso, and macro layers of the environment in effecting change and achieving optimal recovery outcomes (95).

### Future Research

Through the examined literature, it has become clear that the majority of research exploring recovery-oriented practice has been completed using a client-centered approach (5, 96). The paradigm of client-centered mental health care is becoming more widely used and accepted amongst clinicians and researchers (50, 97). Future research using qualitative and quantitative methods must be employed to improve our understanding of recovery from perspectives of family, caregivers, and clinicians. The role of environmental and social factors must also be more carefully considered in future research to facilitate its integration into the design of recovery-oriented interventions. Through further research and continued consideration of the many elements of recovery, clinicians will be better able to engage in meaningful and beneficial recovery-oriented practice (1). Furthermore, critical research into the concept of recovery itself may reinforce the need for substantive restructuring of systems that claim to promote recovery, expanding the focus from the individual to consider cooperative, collective, and systems-level approaches to recovery [(98), p. 145, (99)].

### Limitations

As our search strategy was limited to articles in English, we did not consider articles written in other languages. We also limited the selection of articles to electronic databases and peer-reviewed journal articles available at Queen's University Health Science Library. It is possible that the strategy and inclusion criteria may have limited the number of studies found to be appropriate for the review. We attempted to be comprehensive in our search by employing several strategies: (1) including articles from 1988 (first reference of recovery concept); (2) conducting searches in Google and the six most relevant electronic databases for peer-reviewed articles on recovery and mental health; and (3) consulting a health science librarian and incorporating her input on keywords and search strategy. We also acknowledge that our research team's composition of occupational therapists providing mental health rehabilitation care may have influenced our analysis. Although the team includes one group of stakeholders (occupational therapists seeking to implement recovery-oriented interventions), we could not consult with other key stakeholders (service users and community partners) to validate themes generated, and so did not fully include the sixth stage of scoping review methodology recommended by Levac et al. (100). Engaging clients, family members, caregivers, other mental health professionals or researchers in reviewing this paper and the identified components would have been advantageous. Seeking out explicit feedback through focus groups could identify new ideas or gaps in the paper that could guide future research.

## Conclusions

This scoping review documents important knowledge translation gaps in the literature on recovery elements and identifies a critical need for future studies to address this issue. Our review identified relationships, sense of meaning, and participation as the three major pillars key to recovery for persons with severe mental illness. This review revealed a number of gaps, which may inform future research: (1) lack of standardized elements for conceptualizing recovery for persons with severe mental illness; (2) need to incorporate diversity in perspectives and socio-demographic needs of those with severe mental illness; (3) lack of emphasis on the role of the environment in influencing the process and outcome of recovery. Further research and continued emphasis on the application of the core elements of recovery will facilitate clinicians' engagement in meaningful and effective recovery-oriented practice.

## Data Availability Statement

All datasets generated for this study are included in the article/supplementary material.

## Author Contributions

AJ and KC contributed to the conception and design of the study. AJ and SG reviewed the title and abstracts of all the articles found during the initial search. All authors were involved equally in screening the articles using inclusion and exclusion criteria, extracting the information for data charting, qualitative data synthesis, and writing the first draft of the manuscript. SG prepared the numerical summary and tables. PC, TS, SG, KC, and AJ edited the final manuscript for submission. All authors read and approved the submitted version. All authors contributed to the article and approved the submitted version.

## Conflict of Interest

The authors declare that the research was conducted in the absence of any commercial or financial relationships that could be construed as a potential conflict of interest.
